# The short-term effects of air pollutants on influenza-like illness in Jinan, China

**DOI:** 10.1186/s12889-019-7607-2

**Published:** 2019-10-21

**Authors:** Wei Su, Xiuguo Wu, Xingyi Geng, Xiaodong Zhao, Qiang Liu, Ti Liu

**Affiliations:** 1 School of Management Science and Engineering, Shandong University of Finance and Economics, Jinan, Shandong People’s Republic of China 250014; 2 Jinan Center for Disease Control and Prevention, Jinan, Shandong People’s Republic of China 250014; 3 Shandong Center for Disease Control and Prevention, Shandong Provincial Key Laboratory of Infectious Disease Control and Prevention, Shandong University Institution for Prevention Medicine, Jinan, Shandong People’s Republic of China 250014

**Keywords:** Air pollution, Influenza-like illness, Short-term effects; wavelet coherence analysis, Generalized additive model

## Abstract

**Background:**

There is valid evidence that air pollution is associated with respiratory disease. However, few studies have quantified the short-term effects of six air pollutants on influenza-like illness (ILI). This study explores the potential relationship between air pollutants and ILI in Jinan, China.

**Methods:**

Daily data on the concentration of particulate matters < 2.5 μm (PM 2.5), particulate matters < 10 μm (PM10), sulfur dioxide (SO_2_), nitrogen dioxide (NO_2_), carbon monoxide (CO), and ozone (O_3_) and ILI counts from 2016 to 2017 were retrieved. The wavelet coherence analysis and generalized poisson additive regression model were employed to qualify the relationship between air pollutants and ILI risk. The effects of air pollutants on different age groups were investigated.

**Results:**

A total of 81,459 ILI counts were collected, and the average concentrations of PM2.5, PM10, O_3_, CO, SO_2_ and NO_2_ were 67.8 μg/m^3^, 131.76 μg/ m^3^, 109.85 μg/ m^3^, 1133 μg/ m^3^, 33.06 μg/ m^3^ and 44.38 μg/ m^3^, respectively. A 10 μg/ m^3^ increase in concentration of PM2.5, PM10, CO at lag0 and SO_2_ at lag01, was positively associated with a 1.0137 (95% confidence interval *(CI)*: 1.0083–1.0192), 1.0074 (95% *CI*: 1.0041–1.0107), 1.0288 (95% *CI*: 1.0127–1.0451), and 1.0008 (95% *CI*: 1.0003–1.0012) of the relative risk (RR) of ILI, respectively. While, O3 (lag5) was negatively associated with ILI (RR 0.9863; 95%*CI*: 0.9787–0.9939), and no significant association was observed with NO_2_, which can increase the incidence of ILI in the two-pollutant model. A short-term delayed impact of PM2.5, PM10, SO2 at lag02 and CO, O3 at lag05 was also observed. People aged 25–59, 5–14 and 0–4 were found to be significantly susceptible to PM2.5, PM10, CO; and all age groups were significantly susceptible to SO_2_; People aged ≥60 year, 5–14 and 0–4 were found to be significantly negative associations with O_3_.

**Conclusion:**

Air pollutants, especially PM2.5, PM10, CO and SO_2_, can increase the risk of ILI in Jinan. The government should create regulatory policies to reduce the level of air pollutants and remind people to practice preventative and control measures to decrease the incidence of ILI on pollution days.

## Background

Influenza is a severe respiratory infectious disease caused by the influenza virus, which causes infection of 10~20% of the population, 3~5 million severe illnesses and 29.1~64.6 million deaths worldwide [1]. Influenza-like illness (ILI), a common respiratory syndrome, is an indicator to monitor the influenza activity around the world [1–4]. At the same time, except for influenza virus, some respiratory viruses or bacteria may lead to incidence of ILI, including human adenovirus, respiratory syncytial viruses, and human rhinovirus [5, 6].

It is well documented that air pollution is a leading cause of human morbidity and mortality globally, and it can increase the risk of numerous diseases, including respiratory disease [4, 7–14]. Air pollution includes fine particulate matter with an aerodynamic diameter of 2.5 μm or less (PM2.5), particulate matter with an aerodynamic diameter of 10 μm or less (PM10), sulfur dioxide (SO_2_), nitrogen dioxide (NO_2_), carbon monoxide (CO), and ozone (O_3_). In 2015, ambient particle matter was estimated to be the 6th leading risk factor for the global burden of disease and led to 42.41 million deaths and 103.1 million disability adjusted life-years in the world [15]. Thus, the association between air pollution and disease or deaths, especially respiratory illness, has become an important public health research hotspot in recent years. Although air pollution was not the sole cause of respiratory illness, many epidemiological and experimental studies have shown that there are significant interactions between different types of air pollutants and respiratory illnesses, such as exacerbating respiratory syndrome and increasing respiratory illness transmission [16, 17]. For instance, Nguyen TTN et al. illustrated that there was a strong positive association between all air pollutants other than CO (including PM2.5, PM10, NO_2_, SO_2_ and O_3_) and hospital admissions with respiratory syndrome for Hanoi children aged 0–17 years old [14]. Li YR et al. [18] also demonstrated that the increased concentration of all pollutants except O_3_ (including PM2.5, PM10, NO_2_, SO_2_ and CO) increased the number of hospital outpatients with upper respiratory tract infection aged 0–14 years in Hefei. The experimental results have also shown that air pollutants NO_2_, O_3_ and PM could affect airways through inhalation and exacerbate the susceptibility to and severity of respiratory virus infections [19].

However, few epidemiological studies have examined the effects of all six air pollutants on the incidence of ILI. Xu ZW et al. only showed that PM10 and O_3_ were significantly associated with paediatric influenza in Brisbane, Australia [20]. Liang YJ et al. found that the change in PM2.5 directly influenced the transmission of influenza virus in Beijing [21]. Ali ST et al. found that O_3_ had a statistically significant negative association with influenza transmissibility and CO had a weak positive association [22]. Feng C et al. observed a strong positive relationship between PM2.5 and ILI risk during the influenza season in Beijing, and the most pronounced population was the adult group (age 25–59), followed by young adults (aged 15–24) during the influenza season [7]. In contrast to the above results, Huang L et al. documented a significantly positive association between air pollution (PM2.5, PM10 and NO_2_) and ILI, while there was no association between air pollutants (PM2.5, PM10 and NO_2_) and ILI risk for people aged over 25 in Nanjing [4]. Liu XX found that PM10 and PM2.5 had a significant association with clinical ILI, while SO_2_ and NO_2_ had no relationship [23].

Due to the differences in regions and type of air pollutants, different studies have demonstrated diverse associations between air pollution and ILI. Therefore, it is necessary to assess the association between all six air pollutants and ILI in Jinan, not only PM2.5, including PM10, NO_2_, O_3_, CO and SO_2_.

Jinan is the capital of Shandong Province, China, with an area of 7998 km^2^ comprising 7 zones and 3 counties, with a total population of 6.33 million by the end of 2016 (Jinan Bureau of statistics (2017) [24]. Located on the Lower Yellow River, Jinan is considered a national transportation hub as well as the financial, cultural, and educational centre of eastern China. Due to rapid industrial development and the popularization of automobiles, the concentrations of air pollutants in Jinan are sharply higher than the national average, which is one of the most seriously polluted cities in China. The ILI and air pollutants have obvious seasonality in Jinan, with the peaks of ILI, PM2.5, PM10, NO_2_ and SO_2_ in winter (from December to February) and the peak of O_3_ from May to August [6, 25].

In this study, we aimed to investigate the relationship between air pollutants and influenza-like illness from 3 sentinel hospitals in Jinan and to find the main influencing factor on ILI. We also assessed the significant lag time and susceptible age groups. The results of the study will further provide a better understanding of the adverse health effects of air pollutants.

## Methods

### Data

Daily data of ILI of three national influenza surveillance sentinel hospitals in Jinan from January 1, 2016, to December 31, 2017, were collected from the surveillance system at Shandong Provincial Center for Disease Control and Prevention, including QiLu Children’s Hospital of Shandong University, The No. 4 Hospital of Jinan and The No. 6 Hospital of Jinan (The People’s Hospital of Zhangqiu Area, Jinan) located in different 3 zones and represented about a third of the total population. ILI was defined as an outpatient with an acute respiratory infection syndrome with fever ≥38 °C and cough or sore throat. All patients with a diagnosis of ILI from the outpatient and emergency departments of the three sentinel hospitals were enrolled and then divided into five age groups, 0–4, 5–14, 15–24, 25–59 and ≥ 60 years old, according to the National Influenza Surveillance Project published by National Health Commission of the People’s Republic of China [26]. Although three sentinel hospitals did not enrol all ILI patients in Jinan, daily data of the ILI cases, as an important indicator of influenza surveillance, can reflect the epidemic trends of influenza and is always used to explore the relationship between influenza and relatively influencing factors (National Health commission of the People’s Republic of China, 2009) [27].

Daily data of mean temperature and relative humidity in Jinan between 2016 and 2017 were obtained from the China Meteorological Science Data Sharing Service System (http://data.cma.gov.cn).

Daily data of air pollutants, including PM2.5, PM10, SO_2_, NO_2_, CO, and O_3_, were acquired from Jinan Environmental Monitoring Centre. The daily mean pollutant concentrations were calculated based on the data from twenty-eight fixed-site monitoring stations covering the whole geographical area of Jinan.

### Statistical methods

Descriptive statistical analyses were used to reveal the features of ILI, meteorological data and air pollutants. The Spearman correlation was used to illustrate the association between ILI and climatic variables and the air pollutants.

Similar to many environmental and epidemiological time series data, the time-series data of the air pollutants and ILI are subject to non-stationary prosperities that are complex [4, 21]. To account for these variables, wavelet analysis based on the Monte Carlo methods was used to depict the temporal characteristics of the air pollutants and ILI and investigate the association between them, according to the methods described by Cazelles et al. [28]. Wavelet analysis is a useful tool to explore the association of environmental variables and disease, which can decompose time series data into small time–frequency spaces to identify the key frequencies in different time periods. According to the method described by Grinsted et al. [29], the cross wavelet coherence was used to exploit the correlation, time delay and phase structure between the two time series, air pollutant concentrations and ILI. The cross wavelet angle was used to assess the phase difference between components of the time series. The time delay of the two time series was obtained by calculating the phase difference between the two time series. The cross wavelet coherence analysis was performed by using the MATLAB software package downloaded from http://noc.ac.uk/using-science/corsswavelt-wavelet-coherence.

As the daily number of ILI counts approximately followed the poisson distribution, a generalized additive model (GAM) with a quasi-poisson regression was used to further illuminate the association between air pollutant and daily ILI. In the model, the natural cubic spline functions of non-linear variables, including time trend, mean temperature, and relatively humidity, were introduced into the generalized additive models to exclude the longer and seasonal trends and the potential non-linear association between weather conditions and ILI. Akaile’s Information Criterion (AIC) was used to determine the degree of freedom (*df*) of the smoothing function. The smallest AIC value demonstrates the preferred model. Therefore, based on the AIC value, 6 degrees of freedom was used to control long-term trends and seasonal effects, and 3 degrees of freedom was used to adjust the influence of mean temperature and relative humidity. Day of the week (DOW) was introduced as a variable with values from 1 (Sunday) to 7 (Saturday) to control for the short-term trend. The relative risk (RR) was used to demonstrate the effect of air pollutants on ILI. To understand the characteristics of the lag effect between the air pollutant concentration growth and the incident of ILI, the single-day lag model (from lag0 to lag5) and moving average lag model (from lag01 to lag05) were examined.

The model used is shown as follows:

$$ \log \left[E\left({\mathrm{Y}}_t\right)\right]=\beta {X}_i+{s}_{\left(\mathrm{time},\mathrm{df}\right)}+{s}_{\left(\mathrm{temperature},\mathrm{df}\right)}+{s}_{\left(\mathrm{humidity},\mathrm{df}\right)}+\mathrm{DOW}+\mathrm{intercept} $$

Where.

Y_*t*_ represents the number of ILI counts on day *t*; s and *df* denote the smooth spline function and the degree of freedom, respectively; DOW is an indicator variable indicating day of week; *β* is the regression coefficient for each air pollutant, and X_i_ are the air pollutants such as PM2.5, PM10, O_3_, SO_2_, NO_2_ and CO.

Two-pollutant models combining six air pollutants with the strongest effect were also fitted to evaluate the constancy of individual effects. The variance inflation factor (VIF) was selected to illuminate the multi-collinearity in the models. Variables with VIF values over 5 were not included in the analysis simultaneously because of the multi-collinearity in multivariate analysis [30]. The sensitivity analyses were conducted by varying the degree of freedom of smoothing function for the meteorological factors from 2 to 4 and for controlling for the seasonality from 5 to 9.

The analyses were performed for different age groups (0–4, 5–14, 15–59 and ≥ 60 years old) on the lag day with the strongest effects. All statistical analyses were performed by using R3.1.2 software with the “mgcv” package to fit a time-series model [31]. All statistical tests were two-sided with statistical significance (*p* < 0.05).

## Results

### Description of data

**Fig. 1:**
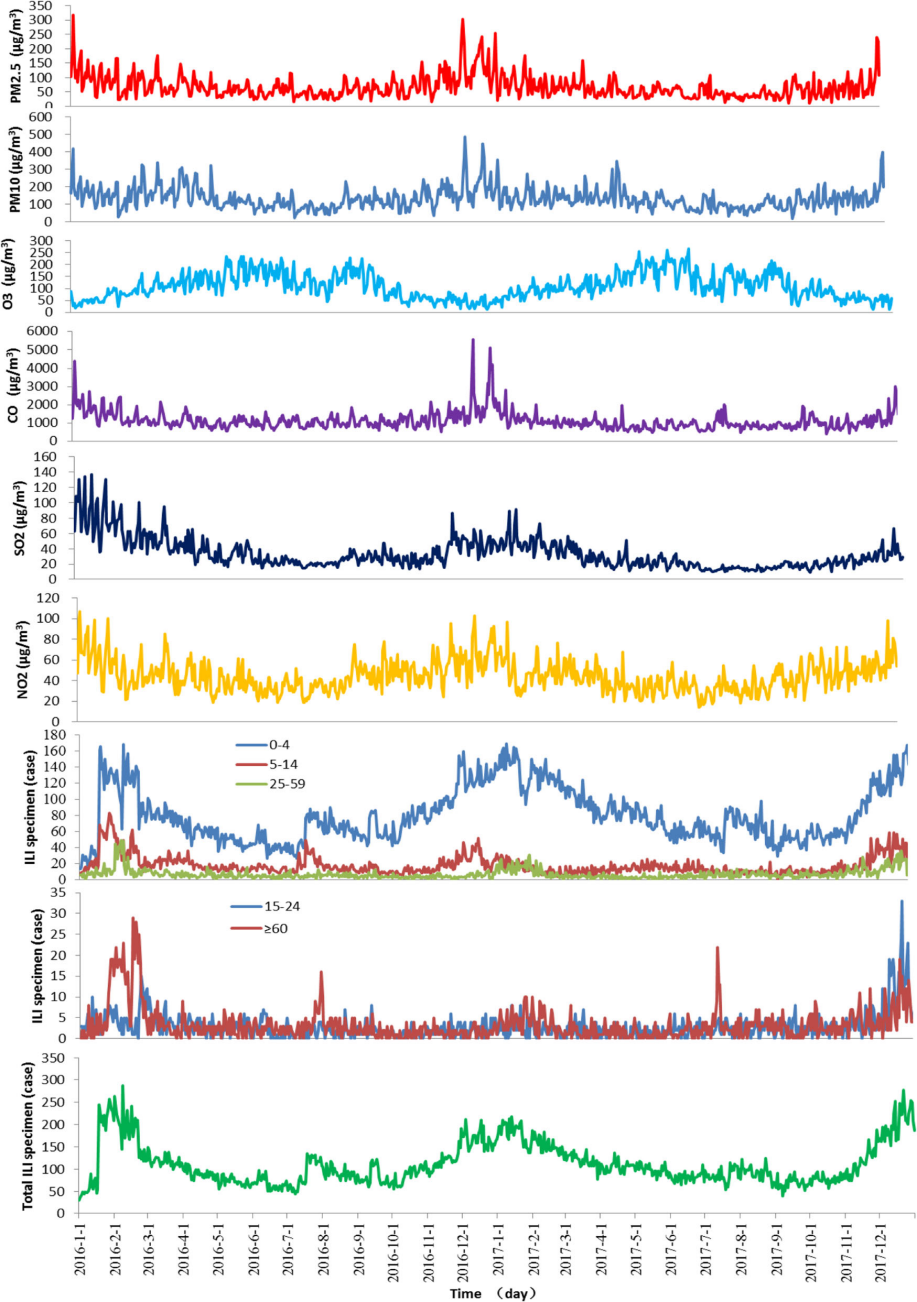
The time-series of air pollutants (daily mean concentration) and ILI incidence (total ILI and different age groups) in Jinan, 2016–2017

A total of 81,459 ILI in Jinan from 2016 to 2017 were enrolled, with the 0–4 year group reporting the most and the 15–24 year group enrolling the least (*P* < 0.001). The descriptive statistics for influenza-like illness, air pollutants and climatic factors are presented in Table 1. Figure 1 presented the time series of air pollutants (daily mean concentration) and the ILI incidence, including total ILI and ILI of different age groups. The daily PM2.5 concentration ranged from 10.24 to 316.25 μg/m^3^, with a daily mean concentration of 67.18 μg/m^3^. The number of days exceeding Grade II China’s National Air Quality Standard (CNAQS) (75 μg/m^3^) was 225 days (30.78% of the study period). The number of days exceeding the Grade II CNAQS of PM10 (150 μg/m^3^), 8 h average O_3_ (160 μg/m^3^), CO (4000 μg/m^3^), SO_2_ (150 μg/m^3^) and NO_2_ (80 μg/m^3^) were 221, 147, 4, 0 and 18 days, respectively. The daily mean air pollutant concentrations did not exceed China’s National Air Quality Standard for residential areas, while all exceeded the WHO guidelines.

**Table 1:** Descriptive statistics for meteorological data, air pollutants and influenza-like illness in Jinan (1 January 2016–31 December 2017)

Meteorological measures	Mean ± S.D	Min	P50	Max
Mean temperature (°C)	15.56 ± 10.305	−12	17.1	33
Relative humidity (%)	55.83 ± 18.71	22	54	98
Air pollutant concentrations				
PM2.5 (μg/m^3^)	67.18 ± 40.18	10.24	56.83	316.25
PM10 (μg/m^3^)	131.76 ± 62.13	16.61	120.7	488
O_3_ (μg/m^3^)	109.85 ± 54.16	11.46	101.48	266.19
CO (μg/m^3^)	1133 ± 510	412	1019	5562
SO_2_ (μg/m^3^)	33.06 ± 19.79	9	26.93	136.61
NO_2_ (μg/m^3^)	44.38 ± 15.49	14.44	42.23	106.82
Daily influenza-like illness				
0–4 years old	80.43 ± 33.18	14	74	169
5–14 years old	17.74 ± 11.79	1	14	83
15–24 years old	2.82 ± 3.10	0	2	33
25–59 years old	7.08 ± 6.33	0	6	49
60 years old and above	3.37 ± 4.05	0	2	29
Total	111 ± 46.83	29	98	287

Note: *S.D* standard deviation

### Spearman’s correlation

The Normality test illustrates that the climatic factors and air pollutants did not fit the normal distribution at the 5% significance level.

Table 2 demonstrates the association of ILI, climatic factors and air pollutants. There were significant positive relationships between ILI and air pollutants, except for O_3_, and significant negative correlations between temperature and relative humidity. Furthermore, the temperature was negatively correlated with air pollutants except O_3_. Except for O_3_, the other air pollutants were positively correlated. The daily mean concentration of PM2.5 was strongly correlated with the daily mean concentration of CO (r = 0.77) and NO_2_(r = 0.66).The daily mean concentration of PM10 was also strongly correlated with the daily mean concentration of CO, SO_2_ and NO_2_.

**Table 2:** Spearson correlation coefficients between ILI, climatic factor and air pollutants in Jinan (1 January 2016–31 December 2017)

Variables	ILI	Mean temperature	Relative humidity	PM2.5/PM10	O_3_	CO	SO_2_	NO_2_
ILI	1							
Mean temperature	−0.62**	1						
Relative humidity	−0.26**	0.26**	1					
PM2.5/PM10	0.31**/0.31**	−0.35**/−0.32**	0.038/− 0.217**	1				
O_3_	−0.51**	0.80**	−0.09*	−0.20**/− 0.13**	1			
CO	0.30**	−0.42**	0.19**	0.77**/0.64**	−0.38**	1		
SO_2_	0.45**	−0.61**	−0.46**	0.59**/0.63**	−0.32**	0.62**	1	
NO_2_	0.31**	−0.54**	−0.13**	0.64**/0.66**	−0.46**	0.74**	0.63**	1

Note: ** *p* < 0.001, * *p* < 0.05

### Wavelet coherence analysis

**Fig. 2:**
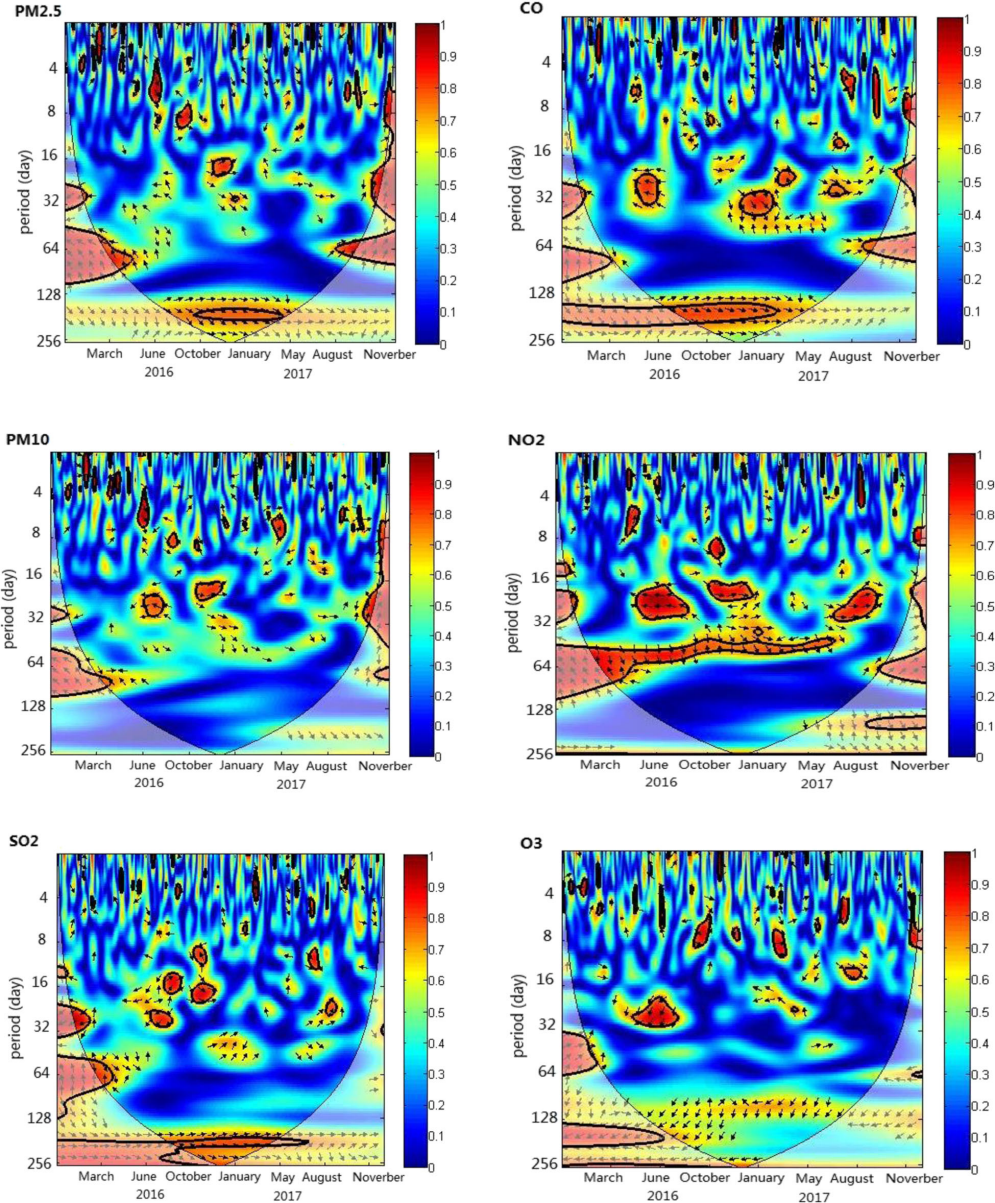
The wavelet coherence of ILI and all air pollutant concentrations. The thick black curve shows the 5% significance level against red noise. The thick black curve shows the 5% significant level against red noise. The cone of influence separating the regions with reliable and less reliable estimates is represented by the lighter pale colors. The color code for power ranges from blue (low power) to red (high power). X-axis denotes the time period and y-axis represents frequencies or scale. The relative phase relationship is shown as arrows (with in–phase pointing right, anti-phase pointing left). Arrows pointing to north-east and south-west mean the X is leading. Arrows pointing to north-west and south-east mean the y is leading

The wavelet coherence analysis was used to illustrate the potential relationship between two time series. Figure 2 indicates the squared wavelet coherence of ILI and air pollutants. For PM2.5, a high power spectrum in the 128–256 day band from November 2016 to April 2017 was observed, with arrows pointing to the right, indicating that the relationship between ILI and PM2.5 was positive. At the same time, there was a significant region at approximately 16–20 days during the 2016–2017 winter seasons, with arrows pointing to the northeast, indicating a delay in ILI following the PM2.5 concentration change of approximately 2 days.

For PM10, we did not observe the phenomena in the 128–256 day band, but a significant region in the approximately 16–20 day band was also observed, indicating a delay in ILI following the PM10 concentration change of approximately 2 days.

Similar to PM2.5, we observed a high power spectrum in the 128–256 day band for SO_2_ and CO, illuminating that the relationship was positive between ILI and SO_2_ or CO. However, we did not observe a significant region to further assess the relative phase and time delay in the time–frequency space between the two time series.

For O_3_, a strong, but not statistically significant, connection at the 5% level during the study period with arrows pointing to the left was observed in the 128–256 day band, showing that the association between ILI and O_3_ was negative.

### Results of regression analysis

**Fig. 3:**
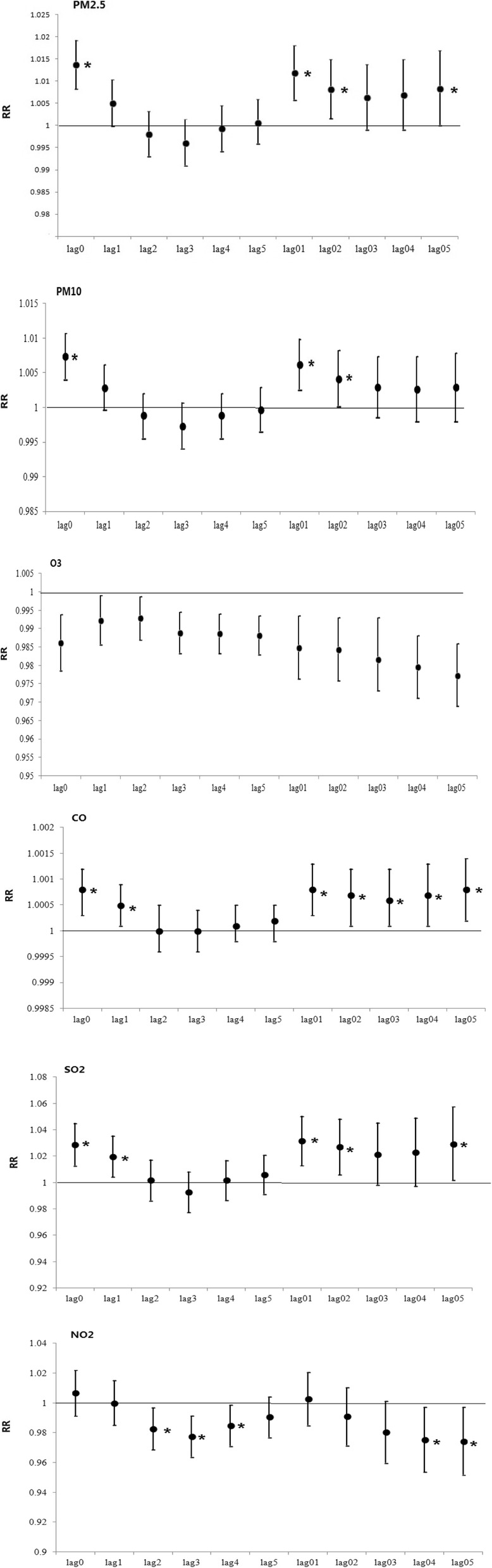
RR change (and 95%CI) of ILI for a 10 μg/m^3^ increase in the levels of the corresponding pollutant on different lag days

Figure 3 demonstrates the percent changes for daily ILI associated with air pollutant concentration at different lag days, including single-day lag (lag0–5) and multi-day lag (lag01–05). For a single-day lag, the effects of air pollutants (PM2.5, PM10, CO and SO_2_) on daily ILI were significant on lag0–1. For multi-day lag, the effects of air pollutants (PM2.5, PM10 and SO_2_) on daily ILI were significant on lag01–02, and CO was on lag01-lag05. For single-day lag and multi-day lag, NO_2_ had no significant relationship with daily ILI. For the air pollutants PM2.5, PM10, SO_2_ and CO, the strongest effects were surveyed on the same day as the day of the hospital visit, and the RRs were 1.0137 (95% CI: 1.0083–1.0192), 1.0074 (95% CI: 1.0041–1.0107), 1.0288 (95% CI: 1.0127–1.0451), and 1.0008 (95% CI: 1.0003–1.0012), respectively. For the cumulative effect of PM2.5, PM10, SO_2_ and CO, the strongest effects were observed on lag01, lag01, lag01 and lag05, with RR values of 1.0119 (95% CI: 1.0058–1.018), 1.0063 (95% CI: 1.0026–1.0099), 1.0315 (95% CI: 1.0129–1.0504), and 1.0008 (95% CI: 1.0002–1.0014), respectively. For O_3_, the strongest impacts for single- and multi-day lags were on lag0 and lag05, with RRs of 0.9863 (95%*CI*: 0.9787–0.9939) and 0.9774 (95%*CI*: 0.969–0.9859), respectively. Table 3 shows the maximum RRs of air pollution affecting ILI in a single-pollutant model.

**Table 3:** Relative risk of air pollutants affecting ILI in a single-pollutant model

Air pollutants	RR	95% CI	*P* value
PM2.5(lag0)	1.0137	1.0083–1.0192	*p* < 0.001
PM10(lag0)	1.0074	1.0041–1.0107	*p* < 0.001
SO_2_(lag01)	1.0315	1.0129–1.0504	*p* < 0.001
CO (lag0)	1.0008	1.0003–1.0012	*p* < 0.001
NO_2_(lag0)	1.0065	0.9913–1.0219	0.404
O_3_(lag05)	0.9774	0.9690–0.9859	*p* < 0.001

Two-pollutant models are presented in Table 4. Considering the co-linearity between PM2.5 and PM10, the two variables did not enrol the same two-pollutant models. The effect estimates dropped by adding other air pollutants to the model except for PM2.5. After the inclusion of the other air pollutants except SO_2_, the effect of PM2.5 at lag0 day increased the statistical significance. In addition, after the inclusion of NO_2_, the RRs of the other air pollutants increased significantly.

**Table 4:** Relative risk (and 95%CI) in daily influenza-like illness in Jinan, China (2016–2017) for a 10 μg/m^3^ increase in the levels of the corresponding pollutant on the lag day with the strongest effects, as estimated from the two-pollutant models

Pollutant	Adjust	RR	95% CI
PM2.5	none	1.0137	1.0083–1.0192
	O_3_	**1.0142**	**1.0088–1.0196**
	CO	**1.0168**	**1.0071–1.0266**
	SO_2_	1.0118	1.0005–1.0184
	NO_2_	**1.023**	**1.0154–1.0306**
PM10	none	1.0074	1.0041–1.0107
	O_3_	1.0074	1.0041–1.0107
	CO	1.0066	1.0013–1.0120
	SO_2_	1.0059	1.0021–1.0096
	NO_2_	**1.0114**	**1.0069–1.0158**
O_3_	none	0.9773	0.9689–0.9858
	PM2.5	0.9785	0.9702–0.9869
	PM10	0.9791	0.9697–0.9865
	CO	0.9774	0.9690–0.9858
	SO_2_	0.9770	0.9687–0.9854
	NO_2_	0.9774	0.9690–0.9859
CO	none	1.0008	1.0003–1.0012
	PM2.5	0.9998	0.9990–1.0005
	PM10	1.0001	0.9995–1.0008
	O_3_	1.0007	1.0003–1.0012
	SO_2_	1.0005	1.000–1.0010
	NO_2_	**1.0016**	**1.0009–1.0022**
SO_2_	none	1.0315	1.0129–1.0504
	PM2.5	1.0142	0.9905–1.0328
	PM10	1.0180	0.9980–1.0385
	O_3_	1.0256	1.0096–1.0418
	CO	1.0196	0.9981–1.0415
	NO_2_	**1.0398**	**1.0177–1.0624**
NO_2_	none	1.0064	0.9913–1.0219
	PM2.5	0.9647	0.9453–0.9845
	PM10	0.9735	0.9543–0.993
	O_3_	1.0041	0.989–1.0193
	CO	0.9629	0.9858–0.9406
	SO_2_	0.9794	0.9599–0.9993

The significance of bold in the table refers to the varaibles with *p* <0.05

**Fig. 4:**
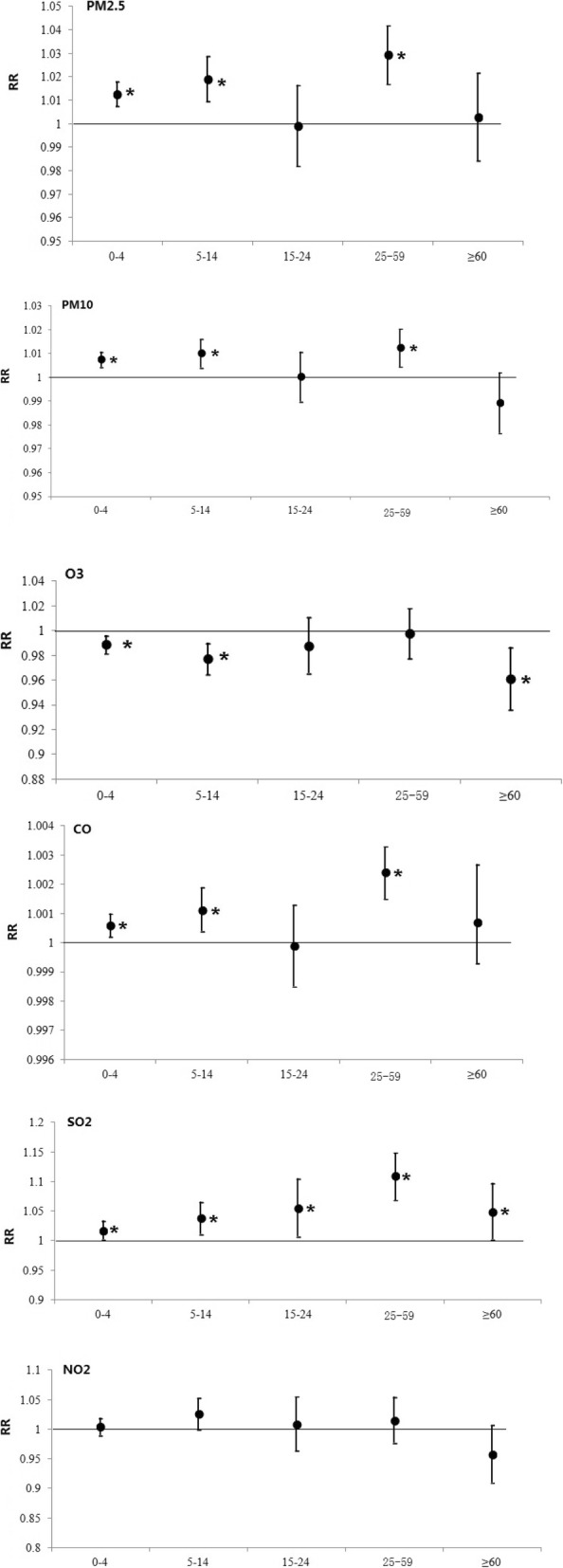
RR change of number of daily ILI cases associated with in a 10 μg/m^3^ increase in the levels of the corresponding pollutant at different age groups on the lag day with the strongest effect in the single-pollutant model

Figure 4 summed up the strongest effects of air pollution on ILI for different age groups in the single-pollutant model. For the effects of PM2.5, PM10 and CO, the RRs for the 10 μg/m^3^ increase were the highest among the 25–59 year group, with 1.0419 (95% CI 1.0168–1.0293), 1.0204 (95% CI 1.0045–1.0124), 1.0033 (95% CI 1.0015–1.0024), respectively, followed by the 5–14 year age group and the 0–4 year age group. For the effects of SO_2_, all age groups were susceptible with the highest RR of the aged 25–59 year group (RR: 1.1495, 95%CI 1.0692–1.1097) and the lowest in the 0–4 year group (RR: 1.0341, 95%CI 1.0019–1.0179). At the same time, O_3_ was found to be more susceptible to people over 60 years of age with RR 0.9866 (95%CI 0.9363–0.9611) compared to the other groups. For NO2, no significant effects were observed in all age groups. For the 15–24 year age group, the results were not significant at the *p* = 0.05 level.

In the sensitivity analyses, we changed the *df* for calendar time from 5 to 9 per year and *df* for climatic factors from 2 to 4. The results suggested that the estimated effects were not substantially changed.

## Discussion and conclusion

Influenza-like illness, as an important hotspot in public health areas, can cause substantial morbidity and mortality each year. Air pollution can increase the incidence of air pollution-related diseases, especially respiratory disease. Previous studies have illuminated that changes in air pollutants can influence the incidence of ILI or respiratory disease [4, 7, 21, 23]. However, different studies provided different results; the cause might be the different study location and the different air pollutants involved. In this study, we brought all six air pollutants into the study and analysed the possible association between the different air pollutants and ILI in Jinan from 2016 to 2017.

With the rapid growth of the population and industry, the increase in PM2.5 concentration in some cities of China is getting unacceptably high, and air pollution has become an extremely critical public health problem in China. In our study, we found that PM2.5 was positively associated with ILI after Spearman’s analysis. The results of the wavelet analysis assessed a potentially positive impact of PM2.5 on daily ILI in the winter, and the change in PM2.5 concentration preceded ILI for two days. After GAM analysis, we further observed that the RR value of ILI was 1.0137 (95% CI: 1.0083–1.0192) and 1.0119 (95% CI: 1.0058–1.018) with a 10 μg/m^3^ increase in PM2.5 concentration on the current day and lag01 days, respectively, which verified the result of wavelet analysis. The observed results were consistent with previous epidemiology studies [4, 23, 32]. PM, a mixture of particles and droplets in the air, can raise the virus attachment to respiratory epithelial cells and deposit deep in the lung due to the small size and larger surface-to-volume ratio. Exposure to PM2.5 not only led to airway epithelial damage and barrier dysfunction but also decreased the ability of macrophages to phagocytize viruses, which raised the susceptibility of an individual to viruses [33].

Although we did not observe a relationship between PM10 and ILI with the cross wavelet approach, we found that PM10 was significantly related to ILI on lag0 and lag02 days through GAM, which was in accordance with previous studies [4, 32, 34] but was different from the relative studies in Hefei for ILI [23] and Hong Kong for hospital admission [35]. One possible reason is that the investigated subjects were different. Liu XX et al. [23] observed changes in weekly ILI, and Wong et al. paid attention to influenza hospitalization cases, while our reports focused on daily ILI [35]. Another reason might be in the linkage of ultraviolet radiation to PM10, which can partly attenuate the effect caused by PM10 [35].

The results of cross wavelet analysis and GAM indicated there was a positive relationship between SO_2_ and ILI cases, which was in accordance with the results of Hwang et al. [34], but different from some previous studies [23, 32]. Some studies have found that the cause of the negative association between SO_2_ and ILI is that the acidic environment influences virus survival and decreases virus transmission [36, 37]. In contrast, an experimental study demonstrated that inhalation of SO_2_ at 26 mg/m^3^ after influenza virus infection can raise the risk of pneumonia in mice [38]. Furthermore, some studies have indicated that SO_2_ can damage the human pulmonary defence system through nonspecific airway reactivity, such as deceasing the mucociliary transpiration rate and alveolar clearance of deposited particles and dysfunction the ability to pulmonary macrophages, which increased the susceptibility to viruses [39]. At the same time, the RR value was the highest in the single-pollutant model, showing people were sensitive to SO_2_, which was in accordance with the previous studies with higher RR or ER values of SO_2_ effects on influenza or other respiratory tract infection [18, 23]. The possible reason might be that the majority of SO_2_ can be dissolved and absorbed easily in the upper respiratory tract, which decreases the immunity of human beings and the resistance to influenza or other respiratory viruses. As for the reason in detail, there need more experimental or epidemiological studies to assessed the effects.

As for the effect of O_3_ on ILI, exposure to O_3_ led to a variety of diverse effects. In our study, the results of both cross wavelet analysis and GAM showed a negative effect of O_3_ on ILI, which was consistent with some previous results [22, 40]. For example, Ali ST et al. illustrated that an increase in O_3_ concentration decreased the transmissibility of influenza virus [22]. However, Wong et al. demonstrated a significantly positive association between O_3_ and pneumonia and influenza admission, with a 10 μg/m^3^ increase resulting in a relative risk of 1.028 (*P* < 0.001), suggesting that exposure to O_3_ would increase the susceptibility to influenza and influenza-related disease [35]. Furthermore, Hwang and Chan et al. and Li YR did not observe an association between respiratory tract illness among children and O_3_ [18, 34]. Because of the diverse results, some studies considered the different concentrations of O_3_ in different cities and different susceptibilities to air pollutants as possible reasons [18]. Some laboratory-based studies have also examined the impacts of O_3_ exposure on respiratory illness. Selgrade et al. [41] found that the mortality of mice infected with influenza virus increased twofold only after day 2 of continuous exposure to 1 ppm O_3_ for 3 h/days, and the mortality had no change on the other days. When the concentration decreased to 0.5 ppm, the effects were not observed. Kesic MJ et al. [42] showed that short exposure to O_3_ (0.4 ppm for 4 h) can enhance influenza virus replication but did not affect the cellular antiviral response. However, Jakab et al. [43] observed that for mice during the course of infection with influenza, exposure to 0.5 ppm O_3_ could reduce the severity of the disease with less widespread infection and decreased pulmonary morbidity. Therefore, the effect of exposure to O_3_ mainly depended on the exposure duration and time during infection. Recent studies have shown that the effects of O_3_ on reduced influenza transmissibility may be associated with O_3_’s virucidal activity and the impact of O_3_ on the host defence [22].

For NO_2_, we did not find any association with daily ILI in either approach, which was consistent with the results of Liu XX in Hefei [23], XZZ in Brisbane [20]. However, Huang et al. [4] and Wong et al. [44] found that NO_2_ was significantly associated with ILI or influenza. The experimental results suggested that the NO_2_-exposed subjects were more easily to be infected with influenza virus because the ability of macrophage-dependent inactivation of the invading pathogen decreased [45]. However, Goings et al. did not observe the statistically significant effect of NO_2_ exposure on influenza infections [46]. Therefore, it is difficult to find an association between NO_2_ and ILI, which need more epidemiological and experimental studies to examine the relationship.

The analysis of the lag effect of air pollution on the occurrence of daily ILI can help explain the mechanisms behind the association and propose a strategy for the control and prevention of ILI. Similar to previous studies, we observed that air pollutant (PM2.5, PM10, CO) exposure on the current day (lag 0) and SO_2_ (lag01), O_3_ (lag05) had the strongest effect on daily ILI. The results of wavelet analysis and GAM analysis showed that there was a 2-day lag for ILI following PM2.5 or PM10 concentration change, which is consistent with the incubation period of the influenza virus and the previous studies in Beijing [7], Nanjing [4], and Hong Kong [35] but different from Xu’s reports with ten lag days in Brisbane, Australia [20]. For this reason, Xu’s report mainly focused on the impact of air pollutants on influenza virus, but we observed the results of ILI caused by different respiratory viruses. At the same time, the results of GAM also showed a 2-day delay for ILI following PM10 and SO_2_ concentration variance and 5-day delays for CO, but we did not observe the same results from the wavelet coherence analysis.

Epidemiology studies have demonstrated that children and the elderly are more likely to be affected because of their weak immune system under bad circumstances of air quality [47]. However, for air pollutants (PM2.5, PM10, CO and SO_2_), the people aged 25–59 was shown to have a higher risk of ILI compared with the other age groups. Similar results were also found by Feng C et al. in a study of the short-term effects of PM2.5 on ILI in Beijing [7]. Our study also showed air pollutants (PM2.5, PM10 and CO) were strongly associated with ILI risk of the groups aged 5–14 and 0–4 (*p* < 0.001) and SO_2_ was strongly related with ILI risk across all age groups (p < 0.001). This might be because the 25–59 year group is the main work group, who spend more time outdoors than the young and the elderly, thus increasing the incidence of exposure to air pollutants. At the same time, we found a significantly negative relationship between O_3_ and ILI under 4 years old, 5–14 age groups and > 60 age groups, which was consistent with the results of Li YR’ et al. in Hefei [18] and the results of Bono et al. [40] and Wang YY et al. in Shanghai [48]. However, Samoli et al. [49] did not find a relationship between O_3_ and paediatric emergency asthma admissions under 4 years old, but they observed a significant association for the 5- to 14-year-old age group in 2001–2004 in Athens, Greece.

The results of our study have important public health implications. First, air pollutants have become an important public health problem because of their adverse health effects. Our results provide evidence that air pollutants can increase the incidence of ILI, and it is essential to take measures to decrease the level of air pollutants and incidence of ILI, including plant or work site shutdown, reduction in the amount of outside activities, school shutdown, or promotion of the use of personal protective equipment (e.g., respirator). Second, our results quantified the relationship between short-term exposure to air pollution and ILI among different age groups. The estimated percent variation can help monitor the adverse health effects caused by air pollution and further assess the risk factors. Finally, our results highlighted the importance of air quality surveillance, which can publish the relative early warning information in due time and take essential measures to protect people’s health and decrease the incidence of ILI and other related diseases.

There are still several limitations in our study. One limitation was the ILI case data. In the study, we only analysed the ILI counts data from three influenza surveillance sentinel hospitals, instead of all hospitals, which may be reduce the effects of air pollutant on ILI or influenza. The three hospitals continues to monitor the change of ILI, the data has a certain coherence and consistency, and can represent perfectly the influenza trend of Jinan recognized by National Health Commission and World Health Organization, which can be used to exploit the correlation between air pollutants and ILI. The second is that the simple daily averaging method might result in measurement errors for pollutants that have a correspondingly large spatial variability with different air pollutant concentrations among 28 surveillance locations. However, if the surveillance location and surveillance method did not change systematically with time, the exposure measurement data collected can perfectly reveal the association based on territory-wide time-series data of both influenza and air pollutants. The third is that the data were collected from Jinan and over two years, which may not perfectly illustrate the association between air pollutants and ILI; thus, the long-term data and more cities would be added for further study.

Despite these limitations, our results still illuminated that short-term exposure to ambient pm2.5, PM10, SO_2_ and CO may increase the occurrence of ILI and O_3_ can decrease the incidence of ILI in Jinan City, China. Further investigation is needed to expand to other areas in Shandong Province, which can further provide more evidence to explain the association between air pollutants and ILI, build an early warning information system and conduct health risk assessment, and help people take essential measures to protect their health in time.

## Data Availability

The datasets used and analysed during the current study are available from the corresponding author on reasonable request.

## Abbreviations

CNAQS: China’s National Air Quality Standard
CO: Carbon monoxide
ER: Excess risk
GAM: Generalized additive model
ILI: Influenza-like illness
NO_2_: Nitrogen dioxide
O_3_: Ozone
PM10: Particulate matter with an aerodynamic diameter of 10 μm or less
PM2.5: Particulate matter with an aerodynamic diameter of 2.5 μm or less
SO_2_: Sulfur dioxide
WHO: World Health Organization
